# Catching Silent Heart Killers—How Bedside Ultrasound Revealed Hidden Endocarditis: A Case Report

**DOI:** 10.5811/cpcem.49037

**Published:** 2026-03-10

**Authors:** Reshvinder Dhillon, Sarah McMullin

**Affiliations:** University of South Alabama, Department of Emergency Medicine, Mobile, Alabama

**Keywords:** POCUS, pediatrics, endocarditis, bacteremia, case report

## Abstract

**Introduction:**

In this report we highlight the emerging role of pediatric cardiac point-of-care ultrasound (POCUS) in rapidly diagnosing infective endocarditis, using a clinical case as illustration.

**Case Report:**

A six-year-old girl with a known ventricular septal defect presented with worsening respiratory symptoms, fevers, abdominal pain, and decreased oral intake. Initial POCUS, performed by an emergency physician, indicated a suspicious echogenic mass in the right atrium, prompting formal echocardiography. Further imaging and cultures confirmed infective endocarditis due to methicillin-sensitive *Staphylococcus aureus*.

**Conclusion:**

This case underscores the utility of pediatric cardiac POCUS as a rapid bedside diagnostic tool for infective endocarditis in emergency settings, leading to early diagnosis and management. Although POCUS cannot replace comprehensive echocardiography, its immediate availability significantly accelerates diagnosis and management initiation, particularly in pediatric patients with congenital heart conditions who are at increased risk for the condition. Ongoing training and standardized protocols will enhance its efficacy. Clinicians should recognize the strengths and limitations of POCUS, integrating it into broader diagnostic workflows for pediatric infective endocarditis.

## INTRODUCTION

Point-of-care ultrasound (POCUS) has emerged as a transformative tool in pediatric emergency medicine, particularly in the evaluation of cardiac conditions.^1^ Over the past decade, technological advances and increasing physician expertise have expanded its applications from basic cardiac assessments to more complex diagnoses, including the detection of vegetations in infective endocarditis.^3^,^4^ In the context of infective endocarditis, POCUS can be used to detect valvular vegetations, although it is not as comprehensive as transthoracic echocardiography or transesophageal echocardiography.

Pediatric emergency physicians (EP) can reliably detect decreased left-sided systolic function and pericardial effusion using POCUS. Case reports show that pediatric EPs have detected right-sided outflow tract obstruction, aortic root dilatation, and congenital cardiac disease using POCUS. Training for pediatric cardiac POCUS competency is feasible, and cardiac POCUS does not increase the burden of cardiology resources on the pediatric emergency department (ED).^3^,^15^

Point-of-care ultrasound presents unique advantages and challenges. Children generally have better acoustic windows than adults, allowing for clearer visualization of cardiac structures. However, the smaller size of cardiac structures and the need for patient cooperation can make image acquisition more challenging. Additionally, the presence of underlying congenital heart defects, which occur in 0.8–1% of live births, can complicate image interpretation.^1^,^4^

## CASE REPORT

A six-year-old female with a history of unrepaired ventricular septal defect and eczema presented to the ED with a one-week history of worsening respiratory symptoms. Her illness began as mild cough and congestion but progressed to abdominal pain, poor oral intake, and fevers. Two months earlier, cardiology follow-up had documented a small, membranous, pressure-restrictive ventricular septal defect with a left-to-right shunt and trace aortic regurgitation.

**Image 1:**
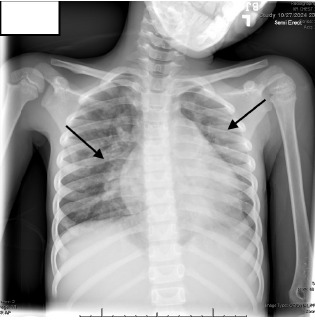
Chest radiograph showing bilateral infiltrate (arrow) in six-year-old child diagnosed with infective endocarditis.

On arrival to the ED, her temperature was 40.4 °Celsius, heart rate 178 beats per minute, respiratory rate 78 breaths per minute, and blood pressure 89/63 millimeters of mercury, with increased work of breathing and a holosystolic murmur. Laboratory studies showed leukocytosis, elevated C-reactive protein, and elevated procalcitonin. A chest radiograph (Image 1) revealed bilateral infiltrates, supporting the ED team’s working diagnosis of pneumonia with sepsis, and she was admitted for antibiotic and respiratory support.

**Image 2:**
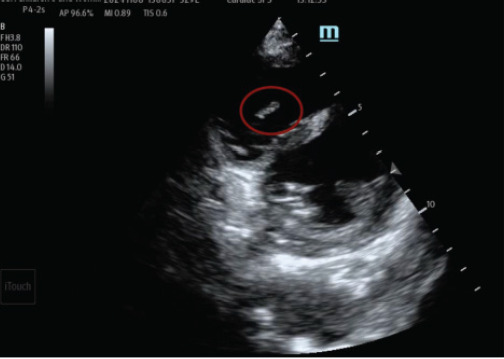
Hyperechoic structure (circled) on the tricuspid valve seen in parasternal short view of a point-of-care ultrasound.

**Image 3:**
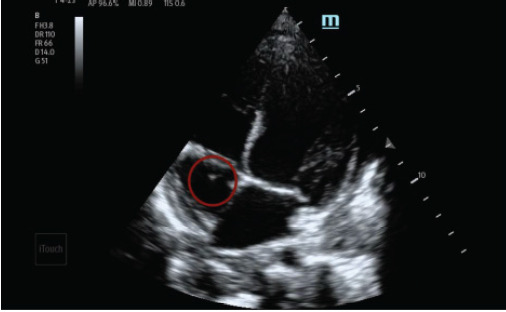
Hyperechoic structure (circled) seen in right atria during systole attached to tricuspid valve in apical four view of a point-of-care ultrasound.

The patient was initially admitted to the pediatric floor but deteriorated rapidly, requiring escalation from high-flow nasal cannula to bilevel positive airway pressure support and transfer to the pediatric intensive care unit (PICU). While in the PICU, an emergency medicine resident rotating on the unit performed a POCUS exam, consistent with institutional training initiatives that encourage residents to integrate POCUS into evaluation of critically ill patients. Although the intent was to assess cardiac function as a possible cause of respiratory distress, the resident unexpectedly identified an echogenic structure in the right atria (Image 2, Image 3). The structure was described as hyperechoic floating between the right atria and the ventricle.

*CPC-EM Capsule*

What do we already know about this clinical entity?

*Infective endocarditis is uncommon in children but risk rises with congenital heart disease; point-of-care ultrasound (POCUS) may show vegetations, yet echo is definitive*.

What makes this presentation of disease reportable?

*Emergency department cardiac POCUS incidentally found a right-sided vegetation in a child presumed septic pneumonia, triggering urgent echo and methicillin-sensitive Staphylococcus aureus infective endocarditis (IE)*.

What is the major learning point?

*Cardiac POCUS can rapidly screen febrile kids with congenital heart disease for mobile echogenic vegetations and speed confirmatory echo/treatment*.

How might this improve emergency medicine practice?

*Using cardiac POCUS in sick children can shorten time to IE recognition, prompt early antibiotics/consults, and allow safe serial reassessment*.

Formal echocardiography confirmed a 16-mm tricuspid valve vegetation consistent with infective endocarditis. Blood cultures and urine cultures were obtained, which subsequently grew methicillin-sensitive *Staphylococcus aureus* (MSSA). Following the MSSA blood culture results and POCUS findings, the patient was started on antibiotic therapy with cefazolin, gentamicin, and rifampin. Gentamicin and rifampin were added for synergistic effect with cefazolin, due to the patient’s critical condition and prolonged bacteremia.

During her 17-day stay in the PICU, the patient developed multiple complications, including septic pulmonary emboli confirmed by computed tomography (CT) angiography. She experienced recurrent episodes of coughing up blood, which required gastrointestinal prophylaxis with famotidine and sucralfate. She also developed iron deficiency anemia that required supplementation. Serial monitoring was performed throughout her admission and included frequent assessments by cardiology with repeat echocardiograms demonstrating persistent but gradually decreasing vegetation size. An ophthalmology evaluation showed no evidence of retinal seeding, and CT of the brain revealed no evidence of cerebral emboli or abscess formation.

The patient completed a 50-day course of intravenous antibiotics via peripherally inserted central catheter line, 50 days of cefazolin and 24 days of rifampin and gentamicin. Final echocardiogram prior to discharge showed a small 6-mm hyperechoic lesion on the septal leaflet of the tricuspid valve, likely representing scarring. This was still present at the four-month cardiology follow-up. She was discharged home with close follow-up with infectious disease, cardiology, and her primary care physician. The patient’s uncontrolled eczema was identified as a possible source of the initial infection, and dermatology follow-up was arranged.

## DISCUSSION

This case highlights the emerging role of POCUS in the early detection of endocarditis in pediatric patients. The rapid identification of a foreign hyperechoic structure by POCUS led to expedited formal imaging and appropriate treatment initiation. In pediatric patients with underlying cardiac conditions presenting with fever or respiratory symptoms, POCUS has become an increasingly valuable initial screening tool.

In suspected infective endocarditis, POCUS can demonstrate vegetations as mobile, irregular, echogenic masses adherent to the valvular surface and moving independently from the valve leaflet. The tricuspid valve is particularly amenable to POCUS due to its anterior position, often best visualized in the apical four-chamber or parasternal short-axis view. In this case, the vegetation’s movement between the right atrium and ventricle was distinctly seen on both views, increasing diagnostic confidence.^7^ Recent studies have demonstrated promising diagnostic accuracy for POCUS in detecting endocarditis. A systematic review by Bai et al found that transthoracic echocardiography, including point-of-care studies, had a pooled sensitivity of 61% and specificity of 94% for detecting vegetations when compared to transesophageal echocardiography.^5^

The diagnostic utility of POCUS in endocarditis varies by vegetation size and location. Vegetations larger than 10 mm are detected with higher sensitivity (> 90%), while those < 5 mm may be missed on initial POCUS examination.^6^,^7^ Right-sided structures, particularly the tricuspid valve, are generally better visualized than left-sided structures due to their anterior location and proximity to the chest wall. Studies have shown that the sensitivity of transthoracic echocardiography, including POCUS, for detecting vegetations in infective endocarditis to be 70–80% right-sided vegetations, such as those on the tricuspid valve. For left-sided vegetations, the sensitivity of transthoracic echocardiography is generally lower, ranging from 50–60%, depending on the valve involved and the quality of imaging.^8^,^9^

Point-of-care ultrasound is particularly valuable in the diagnosis and management of pediatric infective endocarditis due to its ability to provide rapid, bedside cardiac assessment without the delays associated with formal echocardiography. In emergency settings, POCUS can be performed within minutes of presentation, expediting diagnosis by several hours compared with traditional imaging modalities, which is especially critical in pediatric patients in whom early intervention significantly improves clinical outcomes.^10^ Furthermore, POCUS allows for repeat examinations to monitor disease progression. This capability is especially advantageous in critically ill pediatric patients for whom transportation to the echocardiography laboratory may pose clinical risk or instability.^11^

The use of POCUS has also been demonstrated to be cost-effective. Studies have shown that initial screening with POCUS can reduce the need for unnecessary comprehensive echocardiograms in low-risk patients, resulting in substantial healthcare cost savings.^12^ Importantly, early detection and treatment initiation facilitated by POCUS have been associated with reduced morbidity in pediatric endocarditis, particularly in children with underlying structural heart disease.^13^

### Limitations of Point-of-care Ultrasound in Pediatric Endocarditis

Despite these advantages, several limitations of POCUS must be acknowledged. The diagnostic accuracy of POCUS is highly dependent on operator skill and experience in cardiac imaging. Evidence demonstrates that accuracy improves with training, but novice users may miss subtle pathological findings.^13^ Additionally, the quality of POCUS images can be limited by patient-related factors such as respiratory motion, body habitus, or inability to remain still, all of which are common challenges in pediatric populations. Finally, while POCUS is highly effective as an initial screening modality, it should be viewed as complementary to comprehensive echocardiography rather than a replacement. Comprehensive echocardiography remains essential for detailed evaluation of cardiac valve function, precise measurement of vegetations, and identification of complications such as abscess formation or valvular perforation.^13^

The role of POCUS in pediatric endocarditis continues to evolve, with ongoing research focused on standardizing protocols and improving training methods. Recent technological advances, including artificial intelligence-assisted interpretation and three-dimensional imaging capabilities, may further enhance the diagnostic accuracy of POCUS in the future.^14^

## CONCLUSION

Point-of-care ultrasound proved to be a valuable tool in the early diagnosis of tricuspid valve endocarditis in this pediatric patient. This case adds to the growing body of evidence supporting the use of POCUS in pediatric emergency medicine for rapid bedside diagnosis of cardiac conditions.^2^,^4^

## Supplementary Information

Video Hyperechoic structure seen in right atria attached to the tricuspid valve in apical four view of a point-of-care ultrasound.
